# Endometrial thickness, Caucasian ethnicity, and age predict clinical pregnancy following fresh blastocyst embryo transfer: a retrospective cohort

**DOI:** 10.1186/1477-7827-7-33

**Published:** 2009-04-22

**Authors:** Michael L Traub, Anne Van Arsdale, Lubna Pal, Sangita Jindal, Nanette Santoro

**Affiliations:** 1 Department of Obstetrics & Gynecology and Women's Health, Albert Einstein College of Medicine and Montefiore Medical Center, 1300 Morris Park Avenue, Mazer Building Room 316, Bronx, NY 10461, USA; 2 Department of Obstetrics, Gynecology & Reproductive Sciences, Yale University School of Medicine, 333 Cedar Street, PO Box 208063, New Haven, CT 06520, USA

## Abstract

**Background:**

In-vitro fertilization (IVF) with blastocyst as opposed to cleavage stage embryos has been advocated to improve success rates. Limited information exists on which to predict which patients undergoing blastocyst embryo transfer (BET) will achieve pregnancy. This study's objective was to evaluate the predictive value of patient and cycle characteristics for clinical pregnancy following fresh BET.

**Methods:**

This was a retrospective cohort study from 2003–2007 at an academic assisted reproductive program. 114 women with infertility underwent fresh IVF with embryo transfer. We studied patients undergoing transfer of embryos at the blastocyst stage of development. Our main outcome of interest was clinical pregnancy. Clinical pregnancy and its associations with patient characteristics (age, body mass index, FSH, ethnicity) and cycle parameters (thickness of endometrial stripe, number eggs, available cleaving embryos, number blastocysts available, transferred, and cryopreserved, and embryo quality) were examined using Student's T test and Mann-Whitney-U tests as appropriate. Multivariable logistic regression models were created to determine independent predictors of CP following BET. Receiver Operating Characteristic analyses were used to determine the optimal thickness of endometrial stripe for predicting clinical pregnancy.

**Results:**

Patients achieving clinical pregnancy demonstrated a thicker endometrial stripe and were younger preceding embryo transfer. On multivariable logistic regression analyses, Caucasian ethnicity (OR 2.641, 95% CI 1.054–6.617), thickness of endometrial stripe, (OR 1.185, 95% CI 1.006–1.396) and age (OR 0.879, 95% CI 0.789–0.980) predicted clinical pregnancy. By receiver operating characteristic analysis, endometrial stripe ≥ 9.4 mm demonstrated a sensitivity of 83% for predicting clinical pregnancy following BET.

**Conclusion:**

In a cohort of patients undergoing fresh BET, thicker endometrial stripe, Caucasian ethnicity, and younger age are positive predictors of clinical pregnancy after fresh BET. These findings may be useful in clinical management of infertile patients undergoing fresh BET cycles.

## Background

Technical advances in assisted reproductive technologies (ART) have led to improving pregnancy rates with in-vitro fertilization (IVF). Traditionally following controlled ovarian hyperstimulation (COH) and oocyte retrieval, cleaving embryos have been transferred 2–3 days after fertilization; over the last decade, improvements in laboratory techniques and embryo culture media have allowed successful in vitro culture of embryos to the blastocyst stage. Blastocyst embryo transfer (BET) has been shown to correlate to higher live birth rates[1]. Higher success rates with BET may reflect an enhanced natural selection process that allows for biologically superior embryos to be transferred, rather than relying on morphological assessment of cleavage stage embryos [2]. Alternatively, improved pregnancy rates with BET may reflect a more synchronous environment between the blastocyst and the endometrium as blastocyst-endometrial interactions are an essential part of implantation [3]. Valbuena et al [4] have suggested that higher detrimental estradiol levels at the time of cleavage embryo transfer compared to the later timing of the blastocyst transfer, may also contribute to the differential pregnancy rates seen. Finally, it has been proposed that decreasing uterine contractility each day following oocyte retrieval may reduce embryo displacement inside the uterus and improve pregnancy success with blastocyst embryos [5].

A large body of retrospective data first suggested higher clinical pregnancy rates after BET. In one of the larger studies, Schwarzler et al. [6] showed clinical pregnancy rates of 44% after blastocyst and 28% after cleavage stage transfer. A prospective, randomized study of single embryo transfer showed high pregnancy rates (33% vs. 23%) after BET in patients with high embryo quality (6–8 cells, <25% fragmentation, and even-numbered blastomeres), proposed to be from natural selection of the best embryos[7]. Papanikolaou et al. [8] in a prospective randomized study showed both higher clinical pregnancy rate (58% vs. 41%) and lower pregnancy loss rate (14% vs. 20%) after single embryo transfer at the blastocyst versus cleavage stage, postulated to be due to lower rates of aneuploidy. A recent Cochrane meta-analysis including nine randomized control trials showed higher live birth rates in blastocyst cycles (OR 1.35, 95% CI 1.05 – 1.74) with no difference in miscarriage or multiple pregnancy rates[1]. The authors suggest this may be due to either natural selection of the 'best embryo' or due to a difference in the interactions between the embryo and uterine environment.

Although these studies have suggested that it is primarily embryological characteristics (i.e. higher number of embryos at the 6–8 cell stage, high quality embryos) that predict the successful development of a blastocyst, a systematic assessment of predictors of successful clinical outcome after BET cycles is lacking. Many articles have examined the ability of embryos to develop to the blastocyst stage and subsequently impact pregnancy rate. Almost none have reported on the ability to differentially predict pregnancy rates once a patient has already developed blastocysts [1]. In one of the larger studies, which examined single blastocyst transfer and where more than 95% of patients received a good quality blastocyst (thus providing a more controlled environment to adjust for embryo factors), no predictive factors were reported within the blastocyst group [8]. In one of the only studies which have reported predictive factors, Richter et al. demonstrated that age, embryo quality, and endometrial thickness were related to clinical pregnancy [9]. Our objective in this study was to identify patient and COH cycle characteristics that may impact on the probability of clinical pregnancy (CP) after fresh BET in our patient population.

## Methods

### Subjects

All IVF cycles between 2003 and 2007 at Montefiore's Institute for Reproductive Medicine and Health (MIRMH) were reviewed. Data were collected from patient charts, computerized ultrasound reports, completed patient intake questionnaires including information on racial and ethnic background, and detailed embryology records. Analyses were restricted to all fresh IVF cycles proceeding to BET (n = 114). IVF cycles utilizing donor oocytes and thawed BET cycles were excluded; ART cycles utilizing pre-implantation genetic screening or diagnosis were included (n = 4). Early follicular phase (cycle days 1–3) serum levels of FSH reflected "ovarian reserve" status [10]; for those patients in whom prior FSH values were available, the highest available level was taken to reflect ovarian reserve for the individual.

Patient management was per routine clinical practice, and all patient stimulation protocols were reviewed and approved by a single clinical director. Ovulation suppression was achieved with either GnRH agonists or antagonists; COH was achieved with injectable gonadotropins and transvaginal ultrasound guided oocyte retrieval was performed 34 hours after hCG injection. Patients met criteria for hCG injection when 2 follicles (mean diameter) were >17 mm. All patients received luteal supplementation with intramuscular progesterone in oil. During the study period, there were no major changes in laboratory personnel or protocols. IVF (n = 48), intracytoplasmic sperm injection (ICSI)(n = 51), and Split cycles (partial IVF/partial ICSI) (n = 15) cycles were included. The decision to proceed to BET was based on the number and cleavage status of available embryos 2 days post insemination; patients with at least six four-cell embryos (<20% fragmentation) that continued to divide were taken to the blastocyst stage. Patients age >35 were additionally taken to blastocyst culture if they failed to achieve CP in a prior ART cycle using cleavage stage embryos (n = 19). Outcome of interest was CP, defined by the presence of an intrauterine gestational sac on trans-vaginal ultrasound. All other cycle outcomes (non-pregnant, biochemical, and ectopic pregnancies) were classified as negative pregnancy (NP).

The study was approved by the Institutional Review Board at Montefiore Medical Center, Bronx, New York. Given the retrospective nature of analyses, written informed consent was waived.

### Statistical analysis

Distribution of continuous variables was assessed by Shapiro-Wilk test. Associations of CP with patient characteristics (age, body-mass index (BMI), FSH, ethnicity) and ART cycle parameters (serum E_2 _levels on day of hCG, maximal cycle thickness of endometrial stripe-ES on day of hCG, number of eggs retrieved, number of mature (metaphase 2) eggs, number of fertilized embryos, available cleaving embryos on Day 2 and 3, number of blastocysts available, transferred, and cryopreserved) were evaluated using Student's T test (for data demonstrating Gaussian distribution) or the Mann-Whitney tests (for skewed data) as appropriate. Spearman's correlation analyses evaluated associations between the various patient and cycle parameters. Fertilization % (% of mature eggs fertilized) by fertilization method (IVF, ICSI, Split) was evaluated by ANOVA. Multivariable logistic regression models reporting odds ratios (OR) ± 95% confidence intervals were created to determine independent predictors of CP following BET as well as to examine the relationship between weight and Caucasian ethnicity. Additionally, the influence of fertilization method on CP was evaluated by chi-squared test (χ^2^). Embryo quality was defined according to our standard assessment practices[11]. Embryo quality was categorized according to the criteria set forth by the Society for Assisted Reproductive Technology (SART) guidelines ('SART Bulletin, 5/7/07) which is currently used for reporting of all SART data. Embryos were assigned as "good" (4AA, 4AB, 4BA, 5AA, 5AB, 5BA, 3AA), "fair" (5AC, 5CA, 4AC, 4CA, 3AB, 3BA, 3BB, 3AC, 3CA, 3BC, 3CB, 3CC) or "poor" (all others). Additionally, a total embryo quality score was calculated by adding up the individual scores of all embryos transferred in a patient (good = 3, fair = 2, poor = 1). Receiver Operating Characteristic (ROC) analyses were used to determine the optimal thickness of ES for predicting CP following BET. A two tailed p <0.05 was considered statistically significant and p values are reported to the third decimal place. All analyses were performed used STATA SE v 8 (College Station, TX).

## Results and discussion

CP was observed in 50% of the BET cycles. Table 1 describes patient and ART cycle characteristics according to cycle outcome. Continuous data are presented as mean ± standard deviation and categorical data are presented as percentage (%). Patients achieving CP compared to NP were significantly younger (32.4 ± 3.5 vs. 34.1 ± 4.1 years old, p = 0.019) but had comparable ovarian reserve status (as reflected by FSH level) and BMI values. The range of patient ages in our cohort was 24 to 43 years. Both groups received a similar number of blastocysts for embryo transfer and had comparable embryological parameters (Table 1). There were no differences in fertilization (%) (p = 0.251) or CP by fertilization method (p = 0.364). For the four cycles utilizing either pre-implantation genetic screening (n = 2, recurrent pregnancy loss) or diagnosis (n = 2, single gene disorders), the genetically normal embryos were either the only blastocysts that grew in culture or they were the best embryos morphologically.

**Table 1:** Patient and cycle characteristics by pregnancy outcome

Characteristic	Clinical Pregnancy	Negative Pregnancy	P-Value
Age (years)^a^	32.4 ± 3.5	34.1 ± 4.1	0.019*
BMI (kg/m2)^b^	24.7 ± 4.2	25.7 ± 5.2	0.573
FSH (mIU/mL)^b^	6.7 ± 2.3	7.1 ± 1.9	0.167
Peak E_2 _(pg/mL)^a, c^	3146.9 ± 1255.9	3498.2 ± 1267.0	0.142
Number of Eggs Retrieved^a^	18.4 ± 6.9	20.4 ± 7.2	0.120
Number of Mature Eggs^b^	15.8 ± 6.5	17.3 ± 6.4	0.176
Number of Eggs Fertilized^b^	11.9 ± 4.6	12.9 ± 4.6	0.199
Number of Day 2 Embryos^b^	11.9 ± 4.3	12.2 ± 4.1	0.573
Number of Day 3 Embryos^b^	10.4 ± 3.8	10.7 ± 3.5	0.608
Number of Blastocysts^b^	6.6 ± 3.3	5.9 ± 2.8	0.435
Endometrial Stripe (mm)^b^	11.2 ± 3.1	10.1 ± 2.6	0.022*
Number of Blastocysts Transferred^b^	2.0 ± 0.6	2.2 ± 0.7	0.349
Number of Blastocysts Cryopreserved^b^	2.3 ± 2.6	1.9 ± 2.4	0.393

^a ^T-Test ^b^Mann-Whitney *U*-test ^c^denotes estradiol value 1 day prior to oocyte retrieval

* denotes statistical significance; Data expressed as mean ± SD with n = 57 in each group.

**Figure 1:**
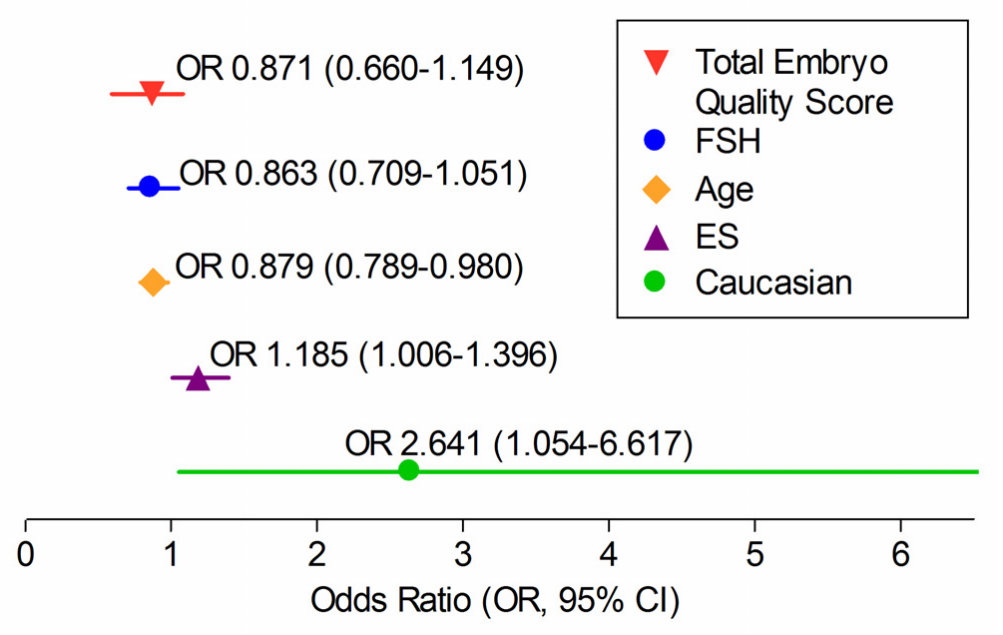
**Multivariable logistic regression analysis showing independent predictors of clinical pregnancy following fresh blastocyst embryo transfer**.

Independent predictors of CP were determined by creating multivariable logistic regression models (Figure 1). Caucasian ethnicity (OR 2.641, 95% CI 1.054–6.617, p = 0.038), thickness of ES (OR 1.185, 95% CI 1.006–1.396, p = 0.042) and age (OR 0.879, 95% CI 0.789–0.980, p = 0.020) significantly predicted CP following BET, after adjusting for FSH and total embryo quality score. Thickness of ES and relationship of cycle outcome with ethnicity were further analyzed to provide more detailed information regarding their predictive values.

**Figure 2:**
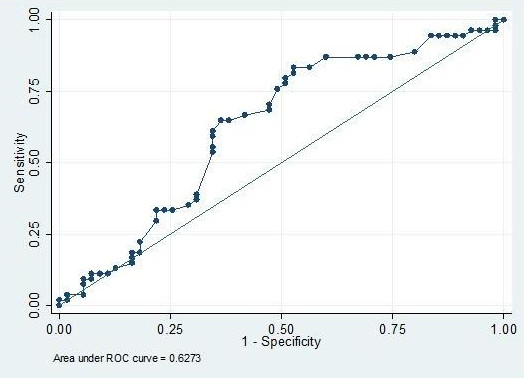
**Receiver-operating characteristic analysis of thickness of ES demonstrates a 9.5 mm cutoff best predicts CP**.

Patients achieving CP had a thicker ES on day of hCG prior to oocyte retrieval (11.2 ± 3.1 vs. 10.1 ± 2.6 mm, p = 0.022) (Table 1). Thickness of ES was not significantly related to patient age (r = -0.093, p = 0.336), BMI (r = 0.117, p = 0.226), FSH (r = 0.056, p = 0.561), or any of the intermediate embryological parameters previously listed (p > 0.05). ROC curves were constructed to evaluate predictive values of thickness of the ES; while an ES thickness ≥ 9.4 mm demonstrated a sensitivity of 83% for predicting CP (Figure 2), area under the ROC curve was modest at 0.627, making the cutoff a poor overall indicator of successful CP (specificity of 47%). Of the 54 clinical pregnancies with known ES thickness, only 3 occurred in patients with an ES thickness <8 mm; the other 9 patients whose ES thickness was <8 mm did not achieve clinical pregnancy.

Data on ethnicity was ascertained from most patient records by the clinician's recorded history (n = 114). The majority of patients in both the CP and NP groups were Caucasian (77% and 64% respectively) whereas non-Caucasians included East Asian, Indian (Indian subcontinent), Hispanic, Black (including African American and African backgrounds), and "other" ethnicity. Caucasian patients yielded significantly fewer blastocyst embryos (5.9 ± 2.9 v 7.3 ± 3.3, p = 0.018) and had fewer embryos available for cryopreservation (1.6 ± 2.3 v 3.3 ± 2.6, p < 0.001) than non-Caucasians, despite comparable ages (p = 0.644), ovarian reserve (p = 0.892), number of prior attempts at IVF (p = 0.408), number of mature eggs (p = 0.678), fertilized embryos (p = 0.913), and day 3 cleavage stage embryos (p = 0.402) (Table 2). Of note, BMI was significantly lower in Caucasian (Table 2) compared to non-Caucasian patients. Multivariable logistic regression analyses adjusting for age identified Caucasians as being significantly less likely to be overweight, i.e. BMI 25–30 kg/m^2 ^(OR 0.304, 95% CI 0.130–0.715, p = 0.006) or obese, i.e. BMI>30 kg/m^2 ^(OR 0.261, 95% CI 0.083–0.827, p = 0.022). On univariate analyses, Caucasian patients were more likely to achieve CP compared to non-Caucasians (55% versus 38%), although the difference was not of statistical significance (chi-square (χ^2^) test, p = 0.151). Additionally, there was still no significant difference in CP when analyzed by individual ethnicity (Fisher's Exact, p = 0.059). However, it is important to note that none of the six Hispanic patients achieved CP (mean age 34.8 years, BMI 32.9 kg/m^2^, FSH 6.7 mIU/mL). Embryo quality was also examined in relation to ethnicity. The total embryo quality score did not differ based on individual ethnicity (Kruskal-Wallis, p = 0.394) or by Caucasian versus non-Caucasian distinction (Mann-Whitney-U test, p = 0.249). Individual ethnicity also did not impact the availability of a "good" quality embryo for transfer (Fisher's exact, p = 0.377). Caucasians were observed to be significantly less likely to have a "good" embryo transferred than non-Caucasians (65% v 85%, chi-square (χ^2^) test, p = 0.029).

**Table 2:** Differences in patient and cycle characteristics by Caucasian ethnicity

Characteristic	Caucasian (n = 80)	Not Caucasian (n = 34)	P-Value
Age (years)^a^	33.4 ± 3.9	33.0 ± 3.8	0.644
BMI (kg/m2)^b^	24.4 ± 3.9	27.1 ± 5.9	0.015*
FSH (mIU/mL)^b^	6.9 ± 2.3	6.9 ± 1.8	0.892
Number of Blastocysts^b^	5.9 ± 2.9	7.3 ± 3.3	0.018*
Endometrial Stripe (mm)^b^	10.6 ± 2.9	10.8 ± 2.8	0.779
Number of Blastocysts Transferred^b^	2.1 ± 0.7	2.0 ± 0.4	0.285
Number of Blastocysts Cryopreserved^b^	1.6 ± 2.3	3.3 ± 2.6	<0.001*

^a ^T-Test ^b^Mann-Whitney *U*-test * denotes statistical significance

Data presented as mean ± SD. Not Caucasian ethnicity included East Asian (n = 7), Indian (n = 12) Black (n = 9), Hispanic (n = 5), and unknown (n = 1) patients.

Finally, we examined the impact of primary etiology of infertility diagnoses on BET cycle outcome. There were no overall differences in contributory infertility etiologies by CP outcome (CP v NP; Fisher's Exact test, p = 0.366). Diagnoses included: male factor (n = 27), tubal factor (n = 28), ovulatory dysfunction (including diminished ovarian reserve; n = 24), unexplained infertility (n = 21), and other causes of infertility (endometriosis, recurrent pregnancy loss, neurofibromatosis, and uterine factors; n = 14). However, infertility diagnosis differed by ethnicity (Fisher's Exact Test, p < 0.001); Caucasians were less likely to have a diagnosis of tubal factor (15% v 66%, p < 0.001) and more likely to have a diagnosis of "other causes of infertility" (18% v 0%, p = 0.009, Fisher's Exact Test).

## Conclusion

In this study, we have identified patient parameters that are predictive of success of fresh BET cycles. Even among this subset of "excellent prognosis" patients by virtue of attainment of blastocyst embryos, advancing age emerged as a detriment to success of BET. This finding supports much evidence in the literature of an overall detrimental effect of aging on oocyte quality and hence, blastocyst biology [12,13].

In addition to age, we identify thickness of ES as an independent predictor of CP following BET; an ES thickness of 9.4 mm or greater was identified as most predictive of successful CP in our patient sample. Prior data relating the significance of ES thickness to pregnancy success after IVF are mixed. Some authors report positive correlations of ES thickness to pregnancy, [14-16], whereas others did not find such associations [17-20]. It is worth noting that the bulk of published literature on relationship between ES thickness and cycle outcome focused on cycles involving transfer of cleaving stage embryos and not blastocysts. Our findings thus extend the previously reported observations on facilitatory implications of thickened ES on IVF cycle outcome to BET cycles and hence provide meaningful addition to the existing body of literature. Additionally those that found significant relationships between ES and pregnancy rates only rarely observed this to be a continuous relationship, as demonstrated in our study; most have identified a threshold thickness of ES as predictive of cycle success. Only 2 authors specifically examined the effect of ES on BET outcome. Zhang et al found that the detrimental effect of a thin ES on pregnancy rates was only applicable to cleavage stage ET and not BET [14]. A single study by Richter et al has specifically identified a positive relationship between thickness of ES and pregnancy after BET [9]; the authors describe a continuous relationship between ES thickness and CP with cutoff values of <9 mm and ≥ 16 mm by ROC analysis; the authors acknowledge poor specificity of ES thickness for cycle outcome, a constraint that is in keeping with our findings.

Approximately 30% of our patients undergoing BET were non-Caucasian, reflecting our diverse ethnic patient population. In our multivariate models, Caucasians were almost three times as likely to achieve CP despite producing fewer blastocysts available for transfer and cryopreservation after adjusting for age, FSH, and number of blastocysts transferred. Other literature supports associations between ethnicity and successful ART outcome, although the exact nature of this association remains elusive [21,22]. Previous disparities in outcome by ethnicity despite similar patient and embryological status have suggested genetic differences as a potential etiology [22].

Of note is the significantly lower BMI in the Caucasian compared to the non-Caucasian population. In the general U.S. population, Caucasian women have a lower BMI and waist circumference compared to African-American and Hispanic women [23,24]. While higher BMI may have causative implications in the reduced likelihood of CP observed in our non-Caucasian patients, we are unable to explore this observed relationship further due to small numbers of patients in each individual non-Caucasian ethnicity. Prior ART literature suggests impaired ovarian responses to COH as well as decreased pregnancy success with increasing BMI [25-27]. However, uterine receptivity was unimpaired in women with increased BMI, when hormonal support and embryo quality were standardized [28,29]. Additionally, Bellver et al. [30] found CP to be unaffected by obesity in oocyte donor-recipient cycles but did see an association between obesity and spontaneous abortion. The existing data imply a link between BMI and oocyte/embryo characteristics that is more likely to account for the reduced clinical pregnancies seen in association with obesity. Interestingly, we found no overall correlation between polycystic ovarian syndrome (PCOS) and BMI (r = 0.024, p = 0.801); additionally PCOS was not correlated with being overweight (r = 0.047, p = 0.620), obese (r = 0.032, p = 0.741), or Caucasian (r = -0.010, p = 0.914). This reinforces that our patient population overall is overweight (mean BMI 25.2 kg/m^2^); only 60/114 (53%) patients had a BMI < 25 kg/m^2^.

A longer duration of infertility prior to treatment may lower pregnancy rates even with BET [31]. Sharara et al. [32] previously found that African-American (compared to Caucasian) women had a significantly longer duration of infertility prior to seeking treatment and had a 3-fold lower odds of achieving pregnancy. Seifer et al. [33] again demonstrated that African-American women had a longer duration of infertility prior to seeking treatment and were underrepresented in IVF centers (5% of cycles) compared to their proportion of the general population of reproductive age women (8%). It is possible that an ethnic disparity in access to ART played a role in our findings. We believe it is especially important for future research to document and systematically study the impact of ethnicity on outcomes of fertility treatments.

Ethnic differences in the prevalence of common conditions associated with infertility may also have played a role in BET success. Ethnic variability in prevalence of both uterine leiomyoma [34] and endometriosis is previously described [35-38] Since the diagnosis of leiomyoma or endometriosis was only represented in our data if identified as the primary cause of infertility, it is not possible to determine whether these conditions varied by ethnicity and/or impacted BET success. The likelihood of a PCOS diagnosis was comparable between Caucasian (11%) and non-Caucasian (12%) patients, a finding common to other published data, [39] and did not seem to impact CP rates in our population.

We acknowledge certain limitations of our study which prevent us from making broader conclusions. The study was retrospective and included a relatively small sample of patients. Ethnicity and thickness of ES though statistically significant were closer to the upper limit of significance which most likely reflects the number of patients included in the study. Our patients were heterogeneous both in their underlying fertility diagnoses and by ethnicity. Therefore broader implications on noted relationships with ethnicity are limited. Both ethnicity and diagnosis impact pregnancy rates; not all diagnoses confer the same prognosis. Information on the duration of infertility is lacking in our patient population.

We herein have identified differences between patients following fresh BET; specifically, our data identify advancing age, endometrial thickness and patient's ethnicity as independent correlates to the success of BET cycle. These data are consistent with the previous finding that patients achieving clinical pregnancy after BET are younger with a thicker ES [9]. More data are needed to determine the exact way in which ethnicity impacts on success of ART following BET. While the past few years have allowed insights into embryo parameters that help identify patients who are likely to achieve embryo growth to the blastocyst stage, our present observations indicate specific host factors that contribute to the success of BET cycles.

## Competing interests

The authors declare that they have no competing interests.

## Authors' contributions

MT participated in the design of the study, data collection, statistical analysis, and drafted the manuscript. AVA helped with the study design and data collection. LP helped with the statistical analysis and helped to draft the manuscript. SJ helped with the design of the study and the preparation of the manuscript. NS helped prepare the manuscript. All authors read and approved the final manuscript.

## References

[B1] Blake DA, Farquhar CM, Johnson N, Proctor M (2007). Cleavage stage versus blastocyst stage embryo transfer in assisted conception. Cochrane Database Syst Rev.

[B2] Guerif F, Le Gouge A, Giraudeau B, Poindron J, Bidault R, Gasnier O, Royere D (2007). Limited value of morphological assessment at days 1 and 2 to predict blastocyst development potential: a prospective study based on 4042 embryos. Hum Reprod.

[B3] Lopata A (1996). Blastocyst-endometrial interaction: an appraisal of some old and new ideas. Mol Hum Reprod.

[B4] Valbuena D, Martin J, de Pablo JL, Remohi J, Pellicer A, Simon C (2001). Increasing levels of estradiol are deleterious to embryonic implantation because they directly affect the embryo. Fertility and sterility.

[B5] Fanchin R, Ayoubi JM, Righini C, Olivennes F, Schonauer LM, Frydman R (2001). Uterine contractility decreases at the time of blastocyst transfers. Human reproduction (Oxford, England).

[B6] Schwarzler P, Zech H, Auer M, Pfau K, Gobel G, Vanderzwalmen P, Zech N (2004). Pregnancy outcome after blastocyst transfer as compared to early cleavage stage embryo transfer. Hum Reprod.

[B7] Zech NH, Lejeune B, Puissant F, Vanderzwalmen S, Zech H, Vanderzwalmen P (2007). Prospective evaluation of the optimal time for selecting a single embryo for transfer: day 3 versus day 5. Fertility and sterility.

[B8] Papanikolaou EG, Camus M, Kolibianakis EM, Van Landuyt L, Van Steirteghem A, Devroey P (2006). In vitro fertilization with single blastocyst-stage versus single cleavage-stage embryos. N Engl J Med.

[B9] Richter KS, Bugge KR, Bromer JG, Levy MJ (2007). Relationship between endometrial thickness and embryo implantation, based on 1,294 cycles of in vitro fertilization with transfer of two blastocyst-stage embryos. Fertility and sterility.

[B10] Broekmans FJ, Kwee J, Hendriks DJ, Mol BW, Lambalk CB (2006). A systematic review of tests predicting ovarian reserve and IVF outcome. Hum Reprod Update.

[B11] Gardner D, Schoolcraft W, Jansen R, Mortimer D (1999). In vitro culture of human blastocysts. Towards Reproductive Certainty: Fertility & Genetics Beyond 1999: the Plenary Proceedings of the 11th World Congress on In Vitro Fertilization & Human Reproductive Genetics.

[B12] Baird DT, Collins J, Egozcue J, Evers LH, Gianaroli L, Leridon H, Sunde A, Templeton A, Van Steirteghem A, Cohen J, Crosignani PG, Devroey P, Diedrich K, Fauser BC, Fraser L, Glasier A, Liebaers I, Mautone G, Penney G, Tarlatzis B (2005). Fertility and ageing. Hum Reprod Update.

[B13] Tatone C, Amicarelli F, Carbone MC, Monteleone P, Caserta D, Marci R, Artini PG, Piomboni P, Focarelli R (2008). Cellular and molecular aspects of ovarian follicle ageing. Hum Reprod Update.

[B14] Zhang X, Chen CH, Confino E, Barnes R, Milad M, Kazer RR (2005). Increased endometrial thickness is associated with improved treatment outcome for selected patients undergoing in vitro fertilization-embryo transfer. Fertility and sterility.

[B15] Kovacs P, Matyas S, Boda K, Kaali SG (2003). The effect of endometrial thickness on IVF/ICSI outcome. Hum Reprod.

[B16] McWilliams GD, Frattarelli JL (2007). Changes in measured endometrial thickness predict in vitro fertilization success. Fertility and sterility.

[B17] Rashidi BH, Sadeghi M, Jafarabadi M, Tehrani Nejad ES (2005). Relationships between pregnancy rates following in vitro fertilization or intracytoplasmic sperm injection and endometrial thickness and pattern. Eur J Obstet Gynecol Reprod Biol.

[B18] De Geyter C, Schmitter M, De Geyter M, Nieschlag E, Holzgreve W, Schneider HP (2000). Prospective evaluation of the ultrasound appearance of the endometrium in a cohort of 1,186 infertile women. Fertility and sterility.

[B19] Puerto B, Creus M, Carmona F, Civico S, Vanrell JA, Balasch J (2003). Ultrasonography as a predictor of embryo implantation after in vitro fertilization: a controlled study. Fertility and sterility.

[B20] Yuval Y, Lipitz S, Dor J, Achiron R (1999). The relationships between endometrial thickness, and blood flow and pregnancy rates in in-vitro fertilization. Hum Reprod.

[B21] Johnson A, El-Toukhy T, Sunkara SK, Khairy M, Coomarasamy A, Ross C, Bora S, Khalaf Y, Braude P (2007). Validity of the in vitro fertilisation league tables: influence of patients' characteristics. Bjog.

[B22] Purcell K, Schembri M, Frazier LM, Rall MJ, Shen S, Croughan M, Grainger DA, Fujimoto VY (2007). Asian ethnicity is associated with reduced pregnancy outcomes after assisted reproductive technology. Fertility and sterility.

[B23] Beydoun MA, Wang Y (2009). Gender-ethnic disparity in BMI and waist circumference distribution shifts in US adults. Obesity (Silver Spring, Md).

[B24] Li C, Ford ES, McGuire LC, Mokdad AH (2007). Increasing trends in waist circumference and abdominal obesity among US adults. Obesity (Silver Spring, Md).

[B25] Fedorcsak P, Dale PO, Storeng R, Ertzeid G, Bjercke S, Oldereid N, Omland AK, Abyholm T, Tanbo T (2004). Impact of overweight and underweight on assisted reproduction treatment. Hum Reprod.

[B26] Fedorcsak P, Storeng R, Dale PO, Tanbo T, Abyholm T (2000). Obesity is a risk factor for early pregnancy loss after IVF or ICSI. Acta Obstet Gynecol Scand.

[B27] Loveland JB, McClamrock HD, Malinow AM, Sharara FI (2001). Increased body mass index has a deleterious effect on in vitro fertilization outcome. J Assist Reprod Genet.

[B28] Styne-Gross A, Elkind-Hirsch K, Scott RT (2005). Obesity does not impact implantation rates or pregnancy outcome in women attempting conception through oocyte donation. Fertility and sterility.

[B29] Wattanakumtornkul S, Damario MA, Stevens Hall SA, Thornhill AR, Tummon IS (2003). Body mass index and uterine receptivity in the oocyte donation model. Fertility and sterility.

[B30] Bellver J, Rossal LP, Bosch E, Zuniga A, Corona JT, Melendez F, Gomez E, Simon C, Remohi J, Pellicer A (2003). Obesity and the risk of spontaneous abortion after oocyte donation. Fertility and sterility.

[B31] Ludwig AK, Diedrich K, Ludwig M (2005). The process of decision making in reproductive medicine. Seminars in reproductive medicine.

[B32] Sharara FI, McClamrock HD (2000). Differences in in vitro fertilization (IVF) outcome between white and black women in an inner-city, university-based IVF program. Fertility and sterility.

[B33] Seifer DB, Frazier LM, Grainger DA (2008). Disparity in assisted reproductive technologies outcomes in black women compared with white women. Fertility and sterility.

[B34] Marshall LM, Spiegelman D, Barbieri RL, Goldman MB, Manson JE, Colditz GA, Willett WC, Hunter DJ (1997). Variation in the incidence of uterine leiomyoma among premenopausal women by age and race. Obstet Gynecol.

[B35] Sangi-Haghpeykar H, Poindexter AN (1995). Epidemiology of endometriosis among parous women. Obstet Gynecol.

[B36] Missmer SA, Hankinson SE, Spiegelman D, Barbieri RL, Marshall LM, Hunter DJ (2004). Incidence of laparoscopically confirmed endometriosis by demographic, anthropometric, and lifestyle factors. Am J Epidemiol.

[B37] Chatman DL (1976). Endometriosis in the black woman. Am J Obstet Gynecol.

[B38] Arumugam K, Templeton AA (1992). Endometriosis and race. Aust N Z J Obstet Gynaecol.

[B39] Azziz R, Woods KS, Reyna R, Key TJ, Knochenhauer ES, Yildiz BO (2004). The prevalence and features of the polycystic ovary syndrome in an unselected population. J Clin Endocrinol Metab.

